# Hormonal therapies for endometriosis-associated pain: inflammatory limitations and pharmacological challenges

**DOI:** 10.1007/s10787-026-02356-6

**Published:** 2026-08-11

**Authors:** Laura García-Izquierdo, Sandra Acedo-Santamaría, Cristina García-Belchí, María Martínez-Esparza

**Affiliations:** Department of Biochemistry and Molecular Biology (B) and Immunology, School of Medicine, University of Murcia, Regional Campus of International Excellence “Campus Mare Nostrum” and Biomedical Research Institute of Murcia (IMIB), Edificio LAIB, 4.54. Avda. Buenavista 32, 30120 El Palmar, Murcia Spain

**Keywords:** Endometriosis, Pain, Hormonal therapy, Inflammation, Pharmacology

## Abstract

Endometriosis is a chronic inflammatory and estrogen-dependent disease in which pain remains the leading cause of impaired quality of life. Hormonal therapies constitute the cornerstone of medical management and include progestins, combined estrogen–progestin contraceptives, gonadotropin-releasing hormone (GnRH) agonists and antagonists, selective estrogen and progesterone receptor modulators, and aromatase inhibitors. Although these treatments effectively suppress ovarian function and reduce estrogen-dependent lesion activity, their clinical benefits are frequently limited by adverse effects, contraceptive implications, and symptom recurrence after treatment discontinuation. Increasing evidence indicates that the persistence of endometriosis-associated pain cannot be explained solely by hormonal dysregulation. A sustained inflammatory microenvironment, characterized by innate immune cell activation, pro-inflammatory cytokine production, and neuroimmune interactions, contributes to peripheral and central sensitization, thereby limiting the effectiveness of therapies targeting endocrine pathways alone. These disease-driven mechanisms provide a biological explanation for the heterogeneous response to hormonal treatment observed in clinical practice. This review summarizes the mechanisms of action, clinical efficacy, safety profile and limitations of current hormonal therapies for endometriosis-associated pain. In addition, it discusses the inflammatory and neuroimmune mechanisms underlying persistent pain and highlights the rationale for combining endocrine therapies with emerging anti-inflammatory and immunomodulatory strategies. Such integrated approaches may improve long-term pain control, reduce recurrence, and contribute to more personalized management of endometriosis.

**Supplementary Information:**

The online version contains supplementary material available at https://doi.org/10.1007/s10787-026-02356-6.

## Introduction

Endometriosis is a chronic inflammatory and estrogen-dependent disorder characterized by the abnormal growth of endometrium-like tissue outside the uterine cavity. It affects approximately 10–15% of women of reproductive age, although accurately determining its prevalence remains a challenge due to delayed diagnosis and clinical heterogeneity (Becker et al. 2021; Giudice et al. 2023). Although dysmenorrhea and chronic pelvic pain (CPP) are the most common symptoms associated with endometriosis, pain intensity does not consistently correlate with disease stage, reflecting the complexity of its underlying mechanisms (Masciullo et al. 2021). CPP significantly impacts quality of life (QoL), affecting physical, emotional, and sexual well-being (ACOG 2020).

Endometriosis-associated pain is increasingly recognized as a multifactorial process involving hormonal, inflammatory, immune, and neurobiological mechanisms. Besides its estrogen dependence, endometriosis is characterized by a persistent inflammatory microenvironment, with increased concentrations of cytokines and other immune mediators in the peritoneal fluid, particularly in advanced stages (Sikora et al. 2018). Elevated levels of pro-inflammatory cytokines, such as interleukin-6 (IL-6) and tumor necrosis factor-α (TNF-α), contribute not only to lesion development but also to peripheral and central sensitization processes (Machairiotis et al. 2021). This complex interplay between hormonal, immune, and neural mechanisms may help explain both the poor correlation between the lesion extent and pain intensity and the heterogeneous response to current hormonal therapies. Although hormonal therapies remain the cornerstone of the pharmacological management of endometriosis-associated pain, they primarily suppress ovarian function without directly targeting the inflammatory and neuroplastic mechanisms that sustain chronic pain, which may contribute to treatment failure, symptom recurrence after treatment discontinuation, and adverse effects that limit long-term use (Ferrero et al. 2026).

The aim of this review is to summarize current hormonal therapies for endometriosis-associated pain, focusing on their mechanisms of action, clinical efficacy, and adverse effects, while discussing their limitations from an inflammatory and pharmacological perspective. In addition, the review examines the inflammatory and neuroimmune mechanisms that may explain the heterogeneous response to hormonal treatment and discusses emerging anti-inflammatory therapeutic strategies that could complement current endocrine approaches.

## Methods

### Literature search strategy

A structured literature search was performed in PubMed to identify original clinical studies evaluating the efficacy and safety of hormonal therapies for the management of endometriosis-associated pain. The search included studies published between January 2000 and June 2026 and was conducted using combinations of free-text terms related to endometriosis, pain, hormonal treatment and adverse effects. The search terms included endometriosis, endometriosis-associated pain, chronic pelvic pain, dysmenorrhea, hormonal therapy, hormonal treatment, side effects, endometriosis drugs and endometriosis pills. Reference lists of eligible articles and relevant reviews were also screened to identify additional studies.

To provide biological context for the discussion of the inflammatory and neuroimmune mechanisms underlying pain persistence and the limitations of hormonal therapies, a complementary narrative review of the literature was performed. Relevant original articles and selected review papers addressing inflammation, immune dysregulation, neuroimmune interactions, pain sensitization, and emerging anti-inflammatory therapeutic strategies in endometriosis were identified through PubMed and by screening the reference lists of relevant publications. These studies were used to support the mechanistic discussion presented in Sect. "Inflammation-driven therapeutic resistance in endometriosis-associated pain".

### Study selection

**Fig. 1:**
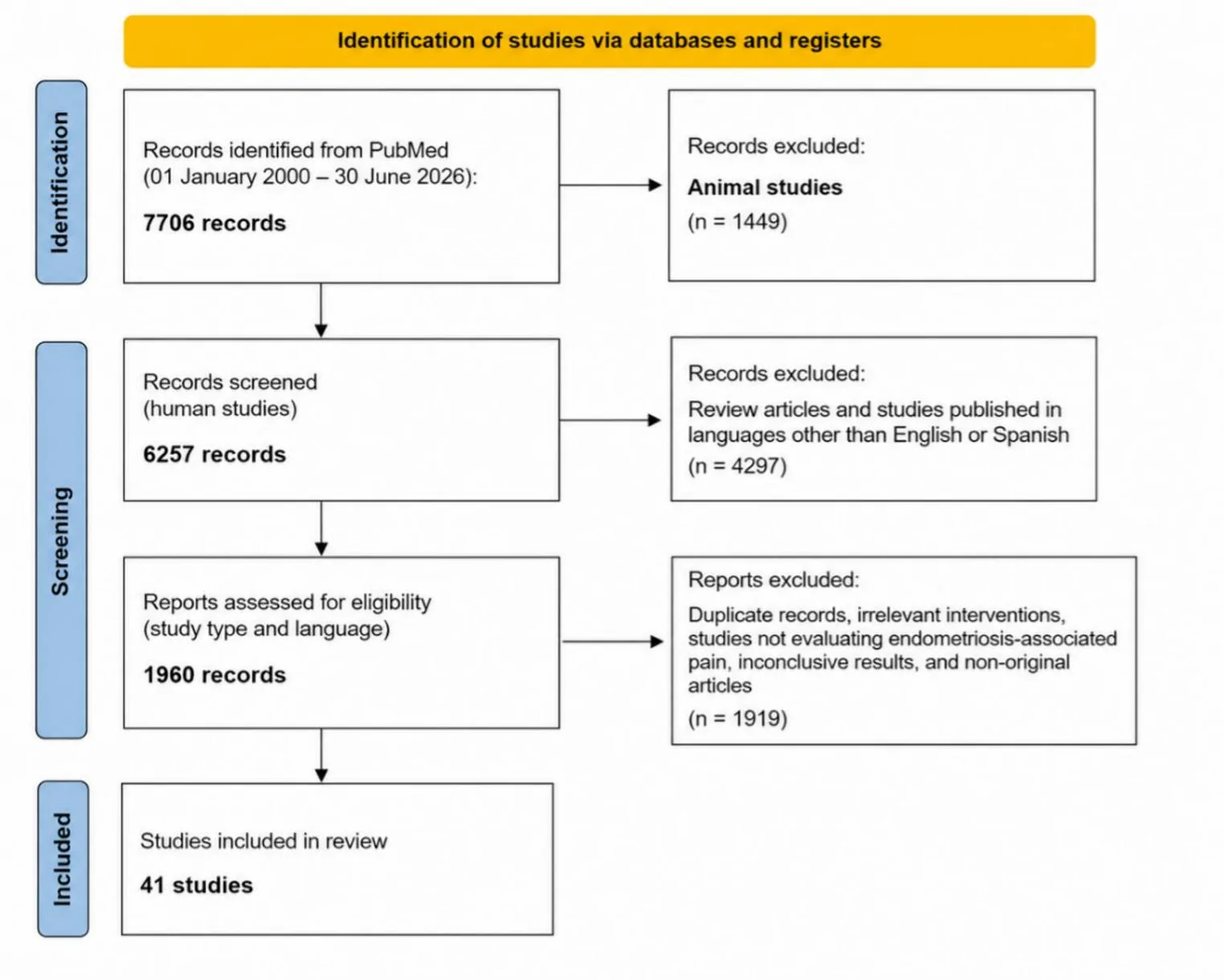
Flow diagram of the literature search and study selection process. Original clinical studies evaluating currently available hormonal therapies for endometriosis-associated pain were identified through a structured PubMed search conducted between January 2000 and June 2026. Records were screened according to predefined eligibility criteria, and studies included in the qualitative synthesis were selected following title, abstract, and full-text assessment. A complementary targeted narrative search was performed separately to support the mechanistic discussion presented in Sect. "Inflammation-driven therapeutic resistance in endometriosis-associated pain" and is therefore not represented in this flow diagram

Titles and abstracts retrieved through the structured search were screened for relevance. Potentially eligible articles underwent full-text evaluation according to predefined inclusion and exclusion criteria. Only original clinical studies evaluating currently available hormonal therapies for endometriosis-associated pain were included in the qualitative synthesis. Eligible study designs comprised randomized controlled trials and prospective or retrospective observational studies. Meta-analyses, review articles, case reports, conference abstracts, editorials, animal studies, and studies lacking sufficient clinical outcome data were excluded from the structured review. The study selection process is summarized in Fig. 1.

To facilitate interpretation of the available evidence, the supplementary tables summarize the design and sample size of the principal clinical studies included for each therapeutic approach.

## Current hormonal therapies

Current management of endometriosis-associated pain combines surgery and pharmacological treatment, depending on symptom severity, disease characteristics, and reproductive goals (Kalaitzopoulos et al. 2021).

**Fig. 2:**
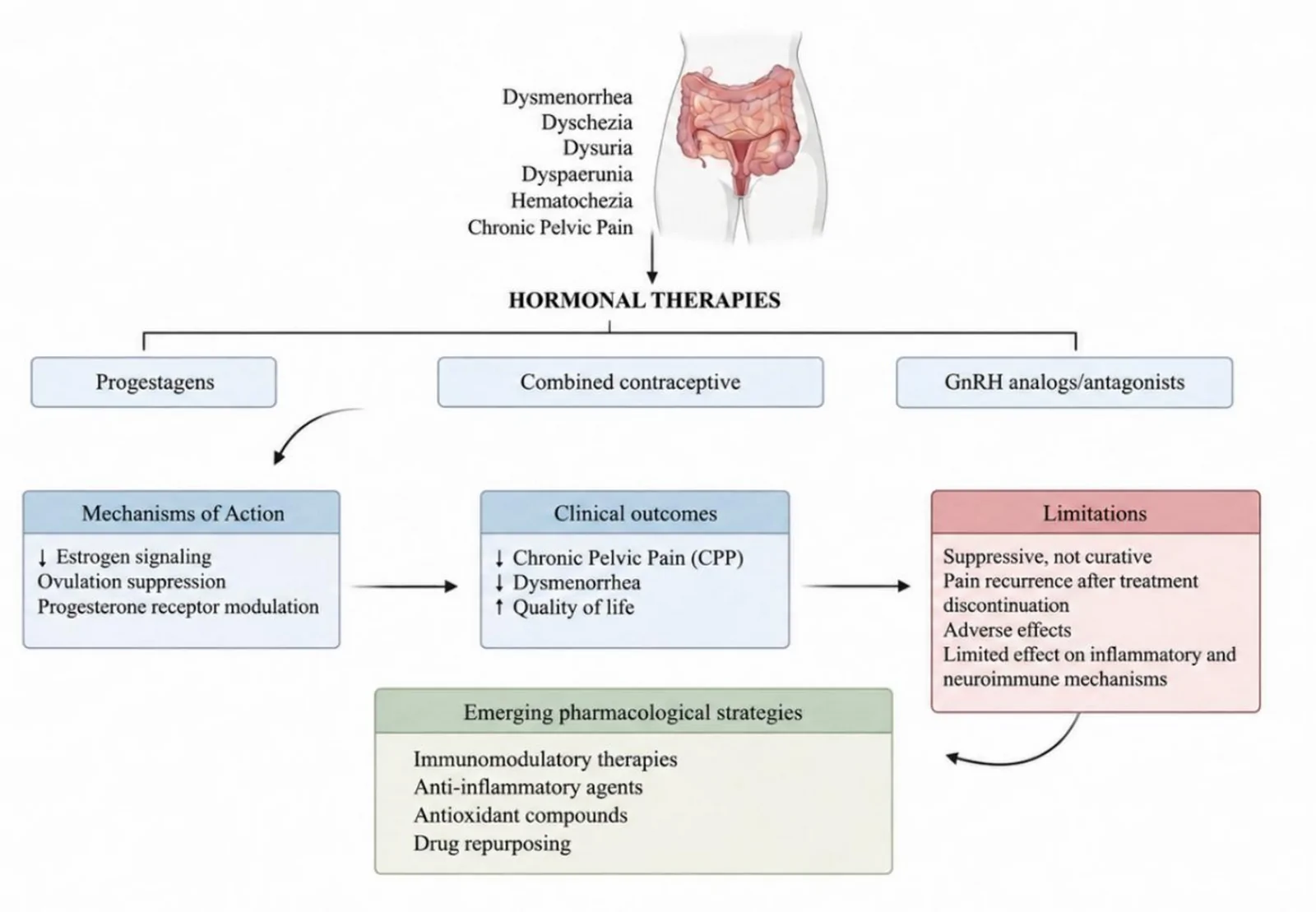
Current hormonal therapies and emerging pharmacological strategies for endometriosis-associated pain. Hormonal therapies, including progestins, combined estrogen–progestin contraceptives, and GnRH analogs/antagonists, primarily suppress ovarian function and reduce estrogen-dependent lesion activity, leading to improvements in CPP, dysmenorrhea, and quality of life. However, these treatments are mainly suppressive rather than curative and are limited by adverse effects, symptom recurrence after treatment discontinuation, and their limited impact on the inflammatory and neuroimmune mechanisms underlying pain persistence. Emerging pharmacological strategies, including immunomodulatory therapies, anti-inflammatory and antioxidant compounds, and drug repurposing approaches, aim to target the inflammatory microenvironment and may complement endocrine therapies to improve long-term clinical outcomes. Created with BioRender.com

Pharmacological management mainly relies on hormonal therapies and nonsteroidal anti-inflammatory drugs (NSAIDs). Hormonal therapies suppress ovarian function or modulate estrogen and progesterone signaling, thereby reducing the growth and activity of endometriotic lesions. Current therapeutic options include progestins, combined estrogen–progestin contraceptives, gonadotropin-releasing hormone (GnRH) agonists and antagonists, selective estrogen receptor modulators (SERMs), selective progesterone receptor modulators (SPRMs), and aromatase inhibitors (AIs) (Fig. 2). Supplementary Tables S1–S5 summarize the principal clinical studies evaluating hormonal therapies. Additional included studies are discussed in the text when they provide complementary information on comparative efficacy, combination therapies, long-term follow-up, safety, or specific clinical observations.

### *Progestogens (*Supplementary Table S1*)*

Nomegestrol acetate binds specifically to the progesterone receptor, exerting strong antiestrogenic effects and potent antigonadotropic activity. Due to its long half-life (50 h), it can cover the hormone-free interval for 4 days. Treatment with nomegestrol acetate is typically combined with 17β-estradiol (E2), resulting in a 24/4 oral contraceptive regimen. Treatment with E2/nomegestrol acetate (1.5 mg/2.5 mg) increased amenorrhea over time while reducing CPP and improving sexual activity and QoL (Caruso et al. 2020).

Dienogest (DNG) is a highly selective progesterone receptor agonist that can be administered continuously without causing major metabolic disturbances. Since its approval in Europe in 2010 for the treatment of endometriosis, oral DNG (2 mg/day) has become one of the most widely used hormonal therapies (Heinemann et al. 2020). Treatment should be maintained for at least 3 months, with 6–12 months generally required to achieve significant reductions in inflammation and endometrioma size. The most common adverse effects include abnormal uterine bleeding, weight gain, headache, and breast tenderness (Cho et al. 2020). Clinical studies have consistently demonstrated that DNG reduces dysmenorrhea, dyspareunia, CPP, endometrioma size, and deep endometriotic lesions while improving QoL (Grandi et al. 2015; Piacenti et al. 2021; Saglik Gokmen et al. 2023). Compared with levonorgestrel/ethinylestradiol, DNG provided greater overall pain relief, whereas both treatments similarly improved dyspareunia, reduced NSAID use, and enhanced QoL (Piacenti et al. 2021). Additional benefits have also been reported when DNG was combined with ethinylestradiol or estradiol valerate, including improvements in dysuria (Del Forno et al. 2023).

In patients newly diagnosed with endometriosis and adenomyosis who were not candidates for surgical treatment, a prolonged flexible oral contraceptive regimen (2 mg of DNG/30 µg of ethinylestradiol) was proposed, consisting of 120 consecutive 30-day cycles of active tablets followed by a 4-day tablet-free interval. A significant decrease in inflammation and in the size of ovarian endometriomas and in uterosacral ligament involvement in adenomyosis was observed at the 12-month follow-up (Carrillo Torres et al. 2023).

Etonogestrel (3-keto-desogestrel), the active metabolite of desogestrel, is administered as a 68-mg subdermal implant (Nexplanon® or Implanon®) that inhibits ovulation for up to 3 years. Clinical studies have shown significant reductions in dysmenorrhea, dyspareunia, and CPP, together with improvements in QoL, including physical pain, general health, vitality, social functioning, and mental health (Sansone et al. 2018). Niu et al. (2021) conducted a 24-month trial in which 66 patients experienced complete remission of CPP. The most common adverse events were vaginal bleeding and menstrual disturbances, together with generally mild systemic side effects, including weight gain, acne, breast tenderness, mood changes, decreased libido, sleep disturbances, constipation, and skin-related symptoms.

The levonorgestrel-releasing intrauterine system (LNG-IUS) continuously releases levonorgestrel (20 µg/day) into the uterine cavity for up to 5 years. Treatment significantly improved endometriosis-associated symptoms within the first 12–18 months, with sustained clinical benefit during follow-up. The most common adverse effects were menstrual irregularities, persistent pelvic pain, and weight gain (Lockhat et al. 2005). In a comparative study, the LNG-IUS and the etonogestrel subdermal implant showed similar efficacy in reducing non-menstrual pelvic pain (NMPP) and dysmenorrhea, while improving QoL without inducing hypoestrogenism (Carvalho et al. 2018).

Medroxyprogesterone acetate is administered as a 150-mg intramuscular injection every 3 months. In a comparative study with the etonogestrel subdermal implant (Implanon®), both treatments achieved a similar reduction in endometriosis-associated pain (approximately 50%) during the first 3 months, with generally mild and transient adverse effects. After one year, amenorrhea was observed in a similar proportion of women receiving either treatment (14–15%) (Walch et al. 2009). Likewise, postoperative treatment with medroxyprogesterone acetate and combined oral contraceptives showed comparable efficacy in pain control and a similar safety profile (Cheewadhanaraks et al. 2012).

Dydrogesterone has high oral bioavailability and, at relatively low doses, is associated with a favorable safety profile. Unlike other progestins, it does not exert androgenic effects or inhibit ovulation (Schweppe 2009). In the ORCHIDEA study, dydrogesterone administered either cyclically (10 mg two or three times daily from days 5 to 25 of the menstrual cycle) or continuously for 6 months significantly reduced CPP, dysmenorrhea, and analgesic use, while improving sexual well-being and QoL. Uterine bleeding was reported in only 1.1% of participants (Sukhikh et al. 2021).

Danazol has shown limited clinical use because of its unfavorable safety profile. Oral administration (600 mg/day for 6 months) was associated with poor tolerability, with the most frequent adverse effects including weight gain, acne, vaginal bleeding, generalized spasms, vaginitis, pain, hypertonia, and an unfavorable lipid profile characterized by increased LDL and decreased HDL cholesterol, potentially increasing cardiovascular risk (Cheng et al. 2005). In contrast, intrauterine administration of danazol through a danazol-loaded intrauterine device (400 mg for 6 months) effectively reduced dysmenorrhea, pelvic pain, and dyspareunia in women with moderate-to-severe endometriosis while minimizing systemic adverse effects (Cobellis et al. 2004).

### Combined estrogen-progestin contraceptive therapy (Supplementary Table S2)

Combined estrogen–progestin contraceptives are widely used as first-line therapy for women with endometriosis who do not wish to conceive. Their therapeutic effect is based on ovulation suppression and the reduction of estrogen-dependent stimulation of endometriotic lesions.

Treatment with drospirenone (3 mg)/ethinylestradiol (20 µg) stabilized symptom severity and health-related QoL in women with posterior deep infiltrating endometriosis (DIE), while preventing lesion progression, inflammation, and worsening of dysmenorrhea and dyspareunia compared with untreated controls (Mabrouk et al. 2011). No significant differences in symptom relief, lesion progression, or tolerability were observed between continuous and cyclic (24/4) regimens, although intermenstrual spotting and headache were the most common adverse effects (Mabrouk et al. 2012).

Beyond symptom control, combined oral contraceptives may also modulate the immune microenvironment. Treatment with ethinylestradiol/desogestrel reduced macrophage infiltration while increasing NK and Treg cell populations in endometriotic tissue, together with decreased cell proliferation and increased apoptosis in the eutopic endometrium (Waiyaput et al. 2021).

More recently, continuous treatment with estetrol (14 mg)/drospirenone (3 mg) for 6 months significantly reduced CPP and dyspareunia, completely resolved dysmenorrhea through amenorrhea induction, and reduced endometrioma size by approximately 30%, although intermenstrual spotting was the most frequent adverse event (Dell´Aquila et al. 2026).

### Gonadotropin-releasing hormone (GnRH) analogs (Supplementary Table S3)

GnRH agonists suppress ovarian estrogen production through pituitary desensitization and are effective in reducing endometriosis-associated pain. In a comparative study, depot goserelin (3.6 mg every 28 days) and intranasal nafarelin (200 µg twice daily) produced similar reductions in dysmenorrhea, dyspareunia, and CPP, with no significant differences in efficacy. The most common adverse effects were hot flashes, sweating, vaginal dryness, and headache, while nasal irritation was reported only with nafarelin (Bergqvist 2000).

A randomized comparative trial showed that 4 months of triptorelin followed by 8 months of combined estrogen–progestin therapy (gestodene/ethinylestradiol) provided pain relief comparable to that achieved with continuous combined hormonal contraception for 12 months (Parazzini et al. 2000). More recently, a Phase III randomized trial demonstrated that triptorelin acetate administered every 3 months (15 mg) achieved comparable efficacy and safety to the conventional monthly regimen (3.75 mg), while reducing the frequency of injections and maintaining pain relief throughout the 24-week treatment period (Li et al. 2022).

### GnRH Antagonists (Supplementary Table S3)

Elagolix was the first oral GnRH antagonist approved for the management of endometriosis-associated pain. In the ELARIS EM trial, both approved doses (150 mg once daily and 200 mg twice daily) significantly reduced dysmenorrhea and analgesic use, with greater efficacy observed at the higher dose (Taylor et al. 2017). Long-term treatment effectively controlled menstrual pelvic pain while minimizing hypoestrogenic effects, particularly at the lower dose, which was associated with only minimal changes in BMD and may be used for up to 24 months (Abrao et al. 2021; Abbas Suleiman et al. 2020).

Relugolix also demonstrated efficacy comparable to leuprorelin while avoiding the initial hormonal flare associated with GnRH agonists and allowing a faster recovery of menstruation after treatment discontinuation (Osuga et al. 2021). In the SPIRIT 1 and SPIRIT 2 trials, once-daily relugolix combination therapy (relugolix, estradiol, and norethisterone acetate) significantly improved dysmenorrhea, NMPP, and overall endometriosis-related pain, while reducing opioid use and minimizing bone mineral density loss (Giudice et al. 2022).

The EDELWEISS clinical development programme established linzagolix as another effective oral GnRH antagonist. Early studies identified 75 mg/day as the optimal dose to relieve pain while maintaining estradiol concentrations within the therapeutic window and minimizing hypoestrogenic adverse effects (Donnez et al. 2020). Subsequently, the Phase III EDELWEISS 3 trial demonstrated that both 75 mg monotherapy and 200 mg combined with add-back therapy significantly improved dysmenorrhea and NMPP, with the higher dose providing greater symptom control while minimizing vasomotor symptoms and bone loss (Donnez et al. 2024). Long-term extension data confirmed sustained improvements in pain, QoL, dyschezia, dyspareunia, and analgesic use, with only minimal reductions in BMD after 12 months of treatment (Donnez et al. 2026).

Opigolix demonstrated dose-dependent efficacy in reducing overall pelvic pain, dysmenorrhea, and menstrual pelvic pain in the Phase II TERRA study. The treatment was generally well tolerated, with headache, hot flashes, insomnia, tinnitus, and gastrointestinal symptoms representing the most common adverse events (D’Hooghe et al. 2019).

### Selective estrogen receptor modulators (SERMs) (Supplementary Table S4)

Bazedoxifene is a selective estrogen receptor modulator that antagonizes estrogen-induced endometrial stimulation while preserving the beneficial estrogenic effects on bone and the central nervous system. Treatment with bazedoxifene combined with conjugated estrogens reduced menstrual flow and pelvic pain in a patient with stage III endometriosis (Flores et al. 2018). In a single case report, prolonged treatment with bazedoxifene/conjugated estrogens in combination with leuprolide effectively controlled endometriosis-associated pain while reducing the vasomotor symptoms and bone mineral density loss typically associated with GnRH agonists (Hill et al. 2018).

### Selective progesterone receptor modulators (SPRMs) (Supplementary Table S4)

Mifepristone, a progesterone receptor antagonist, has been evaluated as an alternative treatment for endometriosis-associated pain. In a 24-week clinical trial, combination therapy with mifepristone (12.5 mg/day) and gestrinone achieved greater clinical efficacy than gestrinone alone, significantly reducing dysmenorrhea, dyspareunia, pelvic pain, pelvic tenderness, and induration. In addition to pain relief, this combination reduced hormone levels and was associated with improved pregnancy outcomes (Xue et al. 2016).

A retrospective clinicopathological study also reported that prolonged mifepristone exposure may induce morphological changes in ovarian endometriosis that can mimic borderline endometrioid tumors. These findings highlight the importance of careful histopathological interpretation and appropriate clinical correlation, rather than suggesting malignant transformation (Pan et al. 2022).

Ulipristal acetate (15 mg/day) also improved pain symptoms in a patient with treatment-resistant endometriosis; however, treatment was associated with reversible endometrial changes resembling hyperplasia after less than 3 months of therapy (Bressler 2017).

Evidence for both SERMs and SPRMs remains limited compared with progestins and GnRH analogs, highlighting the need for further clinical studies to define their role in the management of endometriosis-associated pain.

### Aromatase inhibitors (AIs) (Supplementary Table S5)

Aromatase inhibitors have been evaluated mainly in combination therapies for women with refractory or severe endometriosis. Anastrozole has demonstrated efficacy both as monotherapy and in combination with oral contraceptives. Combined treatment with anastrozole (1 mg/day) and ethinylestradiol/levonorgestrel provided greater symptom relief than oral contraceptives alone, although pelvic pain exacerbation associated with intermenstrual bleeding was reported in some patients. Adverse effects, including headache, hot flashes, mood changes, and myalgia, were generally mild and resolved during follow-up (Amsterdam et al. 2005). More recently, preoperative treatment with anastrozole (1 mg/day for 6 months) significantly improved dysmenorrhea and CPP while delaying symptom recurrence after surgery (Acién et al. 2021).

Clinical studies with letrozole have focused primarily on combination therapy for rectovaginal endometriosis. Letrozole plus norethisterone acetate was associated with fewer adverse effects, lower treatment discontinuation rates, greater patient satisfaction, and no significant loss of BMD compared with letrozole plus triptorelin. Both regimens significantly reduced pain symptoms, although the reduction in the volume of endometriotic nodules was greater with triptorelin (Ferrero et al. 2011). Likewise, the combination of letrozole with oral contraceptives achieved greater reductions in CPP and dyspareunia than oral contraceptives alone, with a lower incidence of adverse effects (Zhao et al. 2021).

## Inflammation-driven therapeutic resistance in endometriosis-associated pain

Although hormonal therapies effectively suppress ovarian estrogen production and reduce lesion activity, a substantial proportion of women continue to experience CPP despite adequate endocrine suppression (Becker et al. 2022; Giudice et al. 2023). This clinical observation suggests that pain persistence reflects disease-driven inflammatory and neuroimmune mechanisms that progressively become uncoupled from ovarian steroid production rather than true pharmacological resistance (Machairiotis et al. 2021; Sikora et al. 2018). Collectively, these mechanisms provide a biological framework for understanding the heterogeneous clinical response to endocrine therapies and support the development of complementary therapeutic strategies targeting inflammation and neuroimmune dysfunction (Ramírez-Pavez et al. 2021; Wang et al. 2025).

The inflammatory microenvironment characteristic of endometriosis is sustained by the continuous recruitment and activation of innate immune cells within the peritoneal cavity (Ramírez-Pavez et al. 2021; Wang et al. 2025). Among these, macrophages constitute the predominant leukocyte population and play a central role in lesion establishment, angiogenesis, fibrosis, immune dysregulation, and pain generation (Ruiz-Alcaraz et al. 2020; Ramírez-Pavez et al. 2021, 2023; Wang et al. 2025). Endometriotic lesions recruit circulating monocytes that differentiate into activated macrophages under the influence of local cytokines and growth factors. During disease progression, macrophages undergo dynamic changes in their activation profile. Although they exhibit remarkable phenotypic plasticity and encompass a broad spectrum of activation states, endometriosis progression is accompanied by a progressive shift towards M2-like macrophages, resulting in an increased M2/M1 ratio that promotes immune tolerance, angiogenesis, extracellular matrix remodelling and fibrosis while impairing the clearance of ectopic endometrial tissue (Ramírez-Pavez et al. 2021; Kobayashi and Imanaka 2022; Wang et al. 2025).

**Fig. 3:**
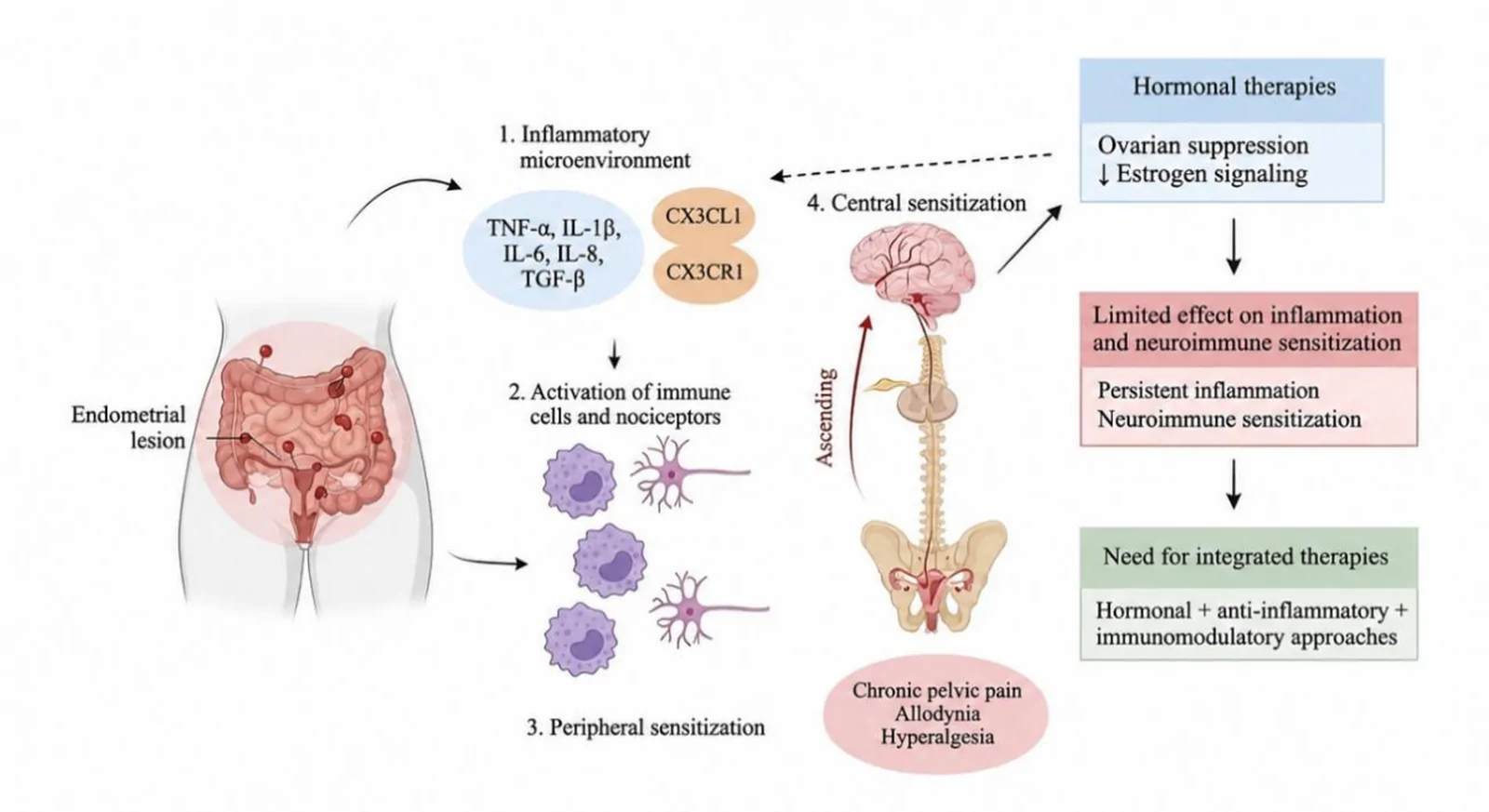
Inflammatory and neuroimmune mechanisms contributing to the limited efficacy of hormonal therapies in endometriosis-associated pain. Endometriotic lesions promote a persistent inflammatory microenvironment characterized by the activation of innate immune cells and the release of pro-inflammatory mediators, leading to peripheral and central sensitization and the maintenance of CPP. Current hormonal therapies primarily suppress ovarian function and reduce estrogen-dependent lesion activity but have limited effects on inflammatory and neuroimmune pathways. Emerging pharmacological strategies targeting these mechanisms may complement endocrine therapies and improve long-term pain control. Created with BioRender.com

These activated immune cells release increased levels of IL-1β, IL-6, TNF-α, transforming growth factor (TGF)-β, prostaglandins, and other inflammatory mediators, thereby contributing to a self-perpetuating inflammatory loop that favours lesion survival while continuously stimulating peripheral nociceptors. In parallel, mast cells accumulate around endometriotic lesions and sensory nerve fibres, where they release histamine, tryptase, prostaglandins, and nerve growth factor (NGF), further amplifying neurogenic inflammation and pain transmission. Neutrophils also contribute during the early stages of lesion development through the release of inflammatory cytokines, reactive oxygen species, and angiogenic factors, facilitating lesion vascularization and maintaining local inflammation (Ramírez-Pavez et al. 2021; Wang et al. 2025). Together, these processes may help explain why inflammatory pathways may remain active despite adequate ovarian suppression, allowing chronic pain to persist in a subset of patients. The mechanisms linking persistent inflammation and neuroimmune activation with the limited efficacy of current hormonal therapies are summarized in Fig. 3.

### Inflammatory and neuroimmune mechanisms sustaining persistent pain

Persistent activation of innate immune cells establishes a complex inflammatory network that extends beyond local tissue injury and plays a central role in pain chronification. Activated macrophages, mast cells, neutrophils, and ectopic endometrial cells release a broad repertoire of cytokines, chemokines, and lipid mediators that interact through multiple positive feedback loops, perpetuating inflammation even in the presence of effective endocrine suppression (Ramírez-Pavez et al. 2021; Wang et al. 2025; Shifon et al. 2025).

Among these mediators, IL-1β, IL-6, TNF-α, and IL-8 promote leukocyte recruitment, amplify cytokine production, and stimulate cyclooxygenase-2 (COX-2)-dependent prostaglandin E2 (PGE2) synthesis, thereby reinforcing inflammatory signalling and nociceptor activation (Burns et al. 2018; Pizzo et al. 2002; Machairiotis et al. 2021). In parallel, TGF-β contributes to tissue remodelling and fibrosis, creating a microenvironment that favours lesion persistence and may further impair normal tissue homeostasis (Hull et al. 2012; Young et al. 2017; Matsuzaki et al. 2022, 2023). Oxidative stress, generated by activated immune cells and iron overload derived from repeated cyclic bleeding, is also thought to amplify these inflammatory pathways by enhancing cytokine production, promoting inflammasome activation, and sustaining chronic tissue damage (Donnez et al. 2016; Machairiotis et al. 2021). Together, these mediators are thought to establish a self-perpetuating inflammatory network that contributes not only to lesion progression but also to the maintenance of persistent pain.

The interaction between immune and nervous system components further amplifies pain signalling through a bidirectional neuroimmune communication network. NGF promotes the sprouting of sensory nerve fibres and increases nociceptor excitability, whereas brain-derived neurotrophic factor (BDNF) contributes to neuronal plasticity and pain sensitization. A recent systematic review and meta-analysis demonstrated increased expression of both neurotrophins in women with endometriosis, supporting their involvement in disease pathophysiology, although evidence for NGF remains limited (Liu et al. 2023). Experimental studies further indicate that endometriotic cells represent an important source of neurotrophins and that reducing NGF and BDNF expression is accompanied by attenuation of macrophage activation and inflammatory signalling, highlighting the close interplay between neurotrophic and immune pathways (Browne et al. 2012; Woo et al. 2019). In addition, the CX3CL1/CX3CR1 signalling axis has emerged as an important mediator of communication between immune cells and sensory neurons, promoting neuroinflammation and sustaining peripheral sensitization (Wang et al. 2025). Macrophages further contribute to neuroimmune crosstalk through the release of inflammatory mediators and insulin-like growth factor-1 (IGF-1), which promotes nerve fibre growth and neuronal sensitization (Greaves et al. 2015; Forster et al. 2019; Wang et al. 2025). These reciprocal interactions are thought to establish a neuroinflammatory microenvironment that reinforces nociceptive signalling and favours pain persistence despite suppression of ovarian hormone production.

Sustained peripheral nociceptor activation eventually induces functional and structural changes within the central nervous system, leading to central sensitization, a process characterized by exaggerated pain responses, allodynia, and hyperalgesia that may persist independently of the initial peripheral stimulus (Machairiotis et al. 2021; Wang et al. 2025). Consequently, although hormonal therapies effectively reduce estrogen-dependent lesion activity, they may be insufficient to reverse the neuroimmune mechanisms responsible for pain chronification once central sensitization has been established. Althought further studies are needed to stablish the clinical contribution of these mechanisms, this concept provides a plausible mechanistic explanation for the heterogeneous clinical response observed among women receiving endocrine therapies and supports the development of complementary therapeutic strategies targeting inflammatory and neuroimmune pathways (Machairiotis et al. 2021; Wang et al. 2025).

### Emerging anti-inflammatory pharmacological strategies

The growing recognition that persistent inflammation and immune dysregulation may contribute to disease progression and pain chronification has prompted the development of novel pharmacological strategies that extend beyond endocrine suppression. Rather than targeting ovarian function, these approaches aim to modulate the inflammatory microenvironment, restore immune homeostasis and interfere with the molecular pathways that sustain lesion persistence, fibrosis and chronic pain (Symons et al. 2018; Saunders and Horne 2021; Chen et al. 2023; Perrone et al. 2025).

Cytokine-targeted therapies have also emerged as promising strategies to complement endocrine treatment by directly modulating the inflammatory microenvironment (García-Izquierdo et al. 2024; Shifon et al. 2025; Perrone et al. 2025). Among these approaches, inhibition of TNF-α, IL-6, IL-1 family signalling, IL-8, CCL2/CCR2 and TGF-β has shown encouraging preclinical results by reducing inflammatory signalling, angiogenesis, immune cell recruitment and lesion growth, although clinical translation remains limited (Shifon et al. 2025). IL-6 has received particular attention owing to its central role in endometriosis pathophysiology and the availability of clinically approved inhibitors for other inflammatory diseases. In experimental models, IL-6 blockade with tocilizumab reduced lesion volume and promoted ectopic endometrial atrophy, although evidence in women with endometriosis is still lacking (Shifon et al. 2025). Likewise, targeting the IL-1 signalling pathway has demonstrated therapeutic potential, with soluble IL-1 receptor type II reducing lesion development in animal models, probably through inhibition of IL-1-induced angiogenesis and IL-6 production (Shifon et al. 2025). Additional experimental evidence indicates that inhibition of the IL-33/MyD88 signalling axis also suppresses lesion growth and cellular proliferation, further supporting IL-1 family members as promising therapeutic targets (Kato et al. 2019). IL-17 has likewise emerged as a potential target, as increased Th17 cells and IL-17 expression contribute to chronic inflammation, lesion progression and immune dysregulation in endometriosis (Kang et al. 2023). Despite this strong biological rationale, most cytokine-targeted therapies remain at the preclinical or early clinical stage, highlighting the need for biomarker-guided patient stratification and precision medicine approaches rather than reliance on single-cytokine blockade (Shifon et al. 2025).

Beyond cytokine-targeted therapies, several additional non-hormonal immunomodulatory strategies are currently being investigated to overcome the inflammatory and immune dysfunction that persists despite endocrine treatment. These include inhibitors of prostaglandin synthesis, antioxidants, inflammasome modulators and therapies aimed at restoring macrophage function (García-Izquierdo et al. 2024; Shifon et al. 2025; Perrone et al. 2025). Among these, macrophage-directed therapies appear particularly attractive because macrophages occupy a central position in the inflammatory, fibrotic and angiogenic networks that drive endometriosis. Restoring macrophage homeostasis may therefore represent a promising strategy to simultaneously reduce inflammation, fibrosis, angiogenesis and lesion progression (Ramírez-Pavez et al. 2021; Wang et al. 2025). Recent studies using macrophage-derived extracellular vesicles and macrophage-targeted nanoparticle delivery systems have further demonstrated that macrophage reprogramming can attenuate disease progression in experimental models, reinforcing its potential as a non-hormonal therapeutic approach (Zhang et al. 2024; Wu et al. 2025).

Increasing attention has also been devoted to drug repurposing as a strategy to accelerate the identification of effective non-hormonal treatments. Several approved drugs with anti-inflammatory, antioxidant or immunomodulatory properties have shown the ability to attenuate inflammatory signalling and reduce lesion progression in experimental studies, highlighting the potential of repositioned compounds as complementary therapies. Nevertheless, their clinical utility in endometriosis-associated pain remains to be established through adequately powered randomized clinical trials.

Overall, current evidence supports the concept that hormonal suppression alone is unlikely to provide optimal long-term symptom control in all patients. Future therapeutic strategies will likely require a more personalized approach combining endocrine therapies with pharmacological interventions targeting inflammation and immune dysregulation. A comprehensive review of emerging pharmacological approaches for endometriosis-associated pain has recently been published; therefore, these strategies are not discussed here in detail (García-Izquierdo et al. 2024).

## Discussion and conclusions

The present review highlights that, despite the availability of several hormonal therapies with different mechanisms of action, their overall efficacy in controlling endometriosis-associated pain is broadly comparable, while long-term disease management remains limited by adverse effects, symptom recurrence after treatment discontinuation, and the inability of endocrine therapies to fully address the inflammatory mechanisms underlying pain persistence (Ferrero et al. 2026).

Importantly, despite differences in their mechanisms of action, the currently available hormonal therapies show broadly comparable efficacy in pain control, and treatment selection is therefore mainly guided by patient characteristics, adverse-effect profiles, treatment duration, contraceptive needs, and reproductive goals.

Long-term management remains challenging because symptom recurrence after treatment discontinuation is common, while adverse effects—including abnormal uterine bleeding, weight gain, headache, vasomotor symptoms, and decreased BMD—may compromise adherence and limit prolonged use. Consequently, no single hormonal therapy is universally suitable for all women, highlighting the need for individualized therapeutic strategies.

The present review also emphasizes that the limited long-term efficacy of hormonal therapies cannot be explained solely by endocrine mechanisms. Increasing evidence indicates that persistent inflammation and neuroimmune dysregulation contribute to pain chronification through biological processes that may remain active despite adequate suppression of ovarian steroid production. Rather than representing true pharmacological resistance, this phenomenon reflects disease-driven mechanisms that are insufficiently targeted by current endocrine therapies.

These observations provide a mechanistic framework for understanding the marked heterogeneity in treatment response observed in clinical practice. They also support the concept that inflammation, immune cell activation, peripheral and central sensitization, and neuroimmune interactions represent complementary therapeutic targets that may improve long-term symptom control when combined with endocrine approaches.

From a translational perspective, future management of endometriosis-associated pain will likely evolve toward more personalized therapeutic strategies integrating hormonal treatments with pharmacological interventions targeting inflammatory and neuroimmune pathways. The identification of biomarkers capable of identifying the predominant pathogenic mechanisms driving pain in individual patients may further facilitate individualized treatment selection and optimize clinical outcomes.

The interpretation of the available evidence should also consider that clinical studies evaluating hormonal therapies differ substantially in study design, patient populations, treatment duration, outcome measures, and sample size. Although randomized controlled trials are available for several therapeutic options, a considerable proportion of the evidence derives from observational studies or relatively small cohorts, which should be considered when interpreting the reported efficacy and generalizability of the findings.

Endometriosis-associated pain is therefore a complex multifactorial condition in which hormonal, inflammatory, immune and neurobiological mechanisms interact to sustain chronic pain and impair quality of life. While hormonal therapies remain the cornerstone of treatment, their integration with immunomodulatory therapies, particularly those targeting macrophage dysfunction, together with antioxidant and anti-inflammatory compounds, and drug repurposing strategies represents a promising avenue for future research rather than an established clinical practice (García-Izquierdo et al. 2024; Ferrero et al. 2026). Although encouraging preclinical evidence is accumulating, robust clinical trials are still required to validate their efficacy and safety before their incorporation into routine clinical management.

The interpretation of the available evidence should also consider that many clinical studies evaluating hormonal therapies differ substantially in study design, patient populations, treatment duration, outcome measures, and sample size. Although randomized controlled trials are available for several therapeutic options, a considerable proportion of the evidence derives from observational studies or relatively small cohorts, which should be taken into account when interpreting the reported efficacy.

Finally, although the evidence supporting current hormonal therapies was identified through a structured literature search, the mechanistic discussion of inflammatory and neuroimmune pathways is based on a complementary narrative review of the literature. This approach was intended to provide biological context for the clinical findings and to facilitate their interpretation rather than to systematically evaluate emerging non-hormonal therapies.

## Supplementary Information

Below is the link to the electronic supplementary material.

Supplementary file1 (DOCX 42 kb)

## Data Availability

No datasets were generated or analysed during the current study.
