# Supplementary material for: Hormonal therapies for endometriosis-associated pain: inflammatory limitations and pharmacological challenges
Source: Inflammopharmacology. 2026 Aug 11;34(9):5919–31. doi: 10.1007/s10787-026-02356-6 (PMC13558358; doi:10.1007/s10787-026-02356-6)
Supplement: Supplementary file 1 [file 10787_2026_2356_MOESM1_ESM.docx]

| **Table S1.** **Progestogens used in the treatment of EM.** | | | | | |
| --- | --- | --- | --- | --- | --- |
| **Drug (class)** | **Study design and participants** | **Dosage and route of administration** | **Beneficial effects** | **Side effects** | **References** |
| **Nomegestrol acetate** | Prospective comparative study  (n=162) | 2.5 mg/day  Oral  6 months | Not evaluated | Potent antigonadotropic and antiestrogenic effects | (Caruso et al. 2020) |
|  |  | 2.5 mg/day  +  E2  Oral  6 months | Amenorrhea, ↓ CPP,  ↑ sexual activity,  ↑ QoL | Not described | (Caruso et al. 2020) |
| **Dienogest** | Prospective cohort study  (n=3356) | 2 mg/day  Oral  6 months | ↓ endometrioma size | Abnormal uterine bleeding, headache, breast tenderness,  ↑ weight | (Cho et al. 2020) |
|  | Prospective observational pilot study  (n=40) | 2 mg/day  +  E2  Oral  6 months | ↓ MPP, ↓ intermenstrual pain, ↓ dyspareunia, = lesion size, ↑ QoL | Headache, bloating, irritability, fatigue | (Grandi et al. 2015) |
|  | Prospective cohort study  (n=80) | 2 mg/day Oral 6 months | ↓ CPP, ↓ dyspareunia,  ↓ endometrioma size, ↓ DIE lesions, ↑ QoL | Vaginal bleeding (mainly during the first 3 months) | (Piacenti et al. 2021) |
| **Etonogestrel** | Multicenter prospective observational study  (n=100) | 68 mg implant  Subcutaneous  12 months | ↓ dysmenorrhea,  ↓ dyspareunia, ↑ QoL | Vaginal bleeding, amenorrhea/oligomenorrhea | (Sansone et al. 2018) |
|  | Prospective observational study  (n=100) | 68 mg implant  Subcutaneous  24 months | ↓ CPP | Weight gain, acne, breast tenderness, mood changes, decreased libido, vaginal bleeding | (Niu et al. 2021) |
| **Levonorgestrel** | Prospective clinical study  (n=100) | 20 µg/day  Intrauterine  5 years | ↓ non-cyclic pelvic pain,  ↓ dysmenorrhea | Menstrual irregularities,  persistent CPP, ↑ weight | (Lockhat et al. 2005) |
|  | Randomized controlled trial  (n=103) | 52 mg LNG-IUS Intrauterine 6 months | ↓ CPP, ↓ dysmenorrhea,  ↑ QoL | Infrequent bleeding, spotting | (Carvalho et al. 2018) |
| **Medroxyprogesterone acetate** | Randomized pilot study  (n=41) | 150 mg/3 months  Intramuscular  12 months | ↓pain | ↑ weight, ↓ libido,  ↓ hair loss, acne, headache,  reversible bone loss | (Walch et al. 2009) |
|  | Randomized comparative trial  (n=84) | 150 mg/3 months  Intramuscular  Comparator: continuous COC  6 months | ↓ dysmenorrhea (greater than COC), ↓ dyspareunia, ↓ NMPP | ↑ weight, oily skin, irritability, amenorrhea/spotting | (Cheewadhanaraks et al. 2012) |
| **Dydrogesterone** | Prospective cohort study  (n=350) | 30 mg/day  Oral  6 months | ↓ dysmenorrhea, ↓ CPP, ↑ sexual well-being,  ↑ QoL | Uterine bleeding | (Sukhikh et al. 2021) |
| **Danazol** | Randomized controlled trial  (n=59) | 600 mg/day  Oral  6 months | ↓ pelvic tenderness,  ↓ induration, ↓ CPP,  ↓ dysmenorrhea,  ↓ dyspareunia | ↑ weight, acne, vaginal bleeding, muscle spasms,  ↓ HDL, ↑ LDL | (Cheng et al. 2005) |
|  | Prospective interventional study (n=18) | 400 mg intrauterine device  Vaginal  6 months | ↓ dysmenorrhea, ↓ CPP,  ↓ dyspareunia | No systemic side effects | (Cobellis et al. 2004) |

*↑* increase; ↓ decrease; = no change; *CPP, chronic pelvic pain; DIE, deep infiltrating endometriosis; E2, estradiol; LNG-IUS, levonorgestrel-releasing intrauterine system; MPP, menstrual pelvic pain; NMPP, non-menstrual pelvic pain; QoL, quality of life.*

| **Table S2.**  **Combination of estrogen and progestin used in EM treatment.** | | | | | |
| --- | --- | --- | --- | --- | --- |
| **Drug (class)** | **Study design and participants** | **Dosage and route of**  **administration** | **Beneficial effects** | **Side effects** | **References** |
| **Ethinylestradiol** | Retrospective observational study  (n=106) | 20 µg/day  +  drospirenone  Oral  21/7 regimen  5.8 months | Stable QoL,  ↓ dysmenorrhea,  ↓ dyspareunia, ↓ lesion progressions | Not described | (Mabrouk et al. 2011) |
|  | Retrospective observational study  (n=93) | 20 µg/day  +  drospirenone  Oral  Continuous or 24/4 regimen  6 months | Similar symptom relief and tolerability with both regimens | Intermenstrual spotting, headache | (Mabrouk et al. 2012) |
|  | Prospective observational study  (n=44) | 30 µg/day  +  desogestrel  Oral  28-35 days | ↓ macrophages, ↑ NK cells, ↑ infiltrating Tregs,  ↓ Inflammation | Not described | (Waiyaput et al. 2021) |
| **Estetrol** | Prospective observational pilot study  (n=31) | 14 mg/day  +  drospirenone  Oral  6 months | ↓ CPP, ↓ dyspareunia,  ↓ dysmenorrhea,  ↑ QoL,  ↓ endometrioma size | Intermenstrual spotting, headache, bloating accompanied by breast tenderness, somnolence | (Dell´Aquila et al. 2026) |

*↑* increase; ↓ decrease; *CPP, chronic pelvic pain; QoL,* quality of life; *NK,* Natural Killer; *Treg,* regulatory T cells.

| **Table S3**. **GnRH used in EM treatment.** | | | | | |
| --- | --- | --- | --- | --- | --- |
| **Analogs** | | | | | |
| **Drug (class)** | **Study design and participants** | **Dosage and route of administration** | **Beneficial effects** | **Side effects** | **References** |
| **Goserelin** | Prospective randomized open-label parallel-group trial(n=113) | 3.6 mg/month  Subcutaneous  2 years | ↓ dysmenorrhea,  ↓ dyspareunia, ↓ CPP | Hot flashes, sweating, vaginal dryness, headache | (Bergqvist 2000) |
| **Nafarelin** | Prospective randomized open-label parallel-group trial  (n=100) | 400 µg/day  Intranasal  2 years | ↓ dysmenorrhea,  ↓ dyspareunia,  ↓ CPP | Hot flashes, sweating, vaginal dryness, headache,  nasal irritation | (Bergqvist 2000) |
|  | Randomized controlled trial  (n=59) | 400 µg/day  Intranasal  6 months | ↓ CPP,  ↓ dysmenorrhea,  ↓ dyspareunia, stable lipid profile | Hot flashes, vaginitis, leukorrhea, menstrual disorders, chest pain | (Cheng et al. 2005) |
| **Triptorelin acetate** | Phase 3 randomized controlled trial  (n=300) | 3.75 mg/month or  15 mg/3 months  Intramuscular  6 months | ↓ CPP | Hot flashes, vaginal bleeding, upper respiratory tract infection, nasopharyngitis, night sweats, abdominal pain, hypersensitivity | (Li et al. 2022) |
|  | Randomized comparative trial  (n=102) | 3.75 mg/month 4 months  Followed by  COC 8 months  Intramuscular  12 months | ↓ NMPP,  ↓ dysmenorrhea | Not described | (Parazzini et al. 2000) |
| **Antagonist** | | | | | |
| **Drug (class)** | **Study design and participants** | **Dosage and route of administration** | **Beneficial effects** | **Side effects** | **References** |
| **Elagolix** | Phase 3 randomized controlled trial  (n=1689) | 150 mg/day  or  200 mg twice/day  Oral  6 months | ↓ dysmenorrhea, ↓ NMPP | Hypoestrogenic effects (hot flashes, ↓ BMD),  lipid changes | (Taylor et al. 2017) |
| **Relugolix** | Phase 2 open-label extension trial  (n=397) | 40 mg/day  Oral  6 months | ↓ pain,  well tolerated | Nasopharyngitis, headache, metrorrhagia, irregular menstruation, hyperhidrosis, hot flashes | (Osuga et al. 2021) |
|  | Phase 3 randomized controlled trial  (n=1261) | 40 mg/day  +  ABT  Oral  6 months | ↓ dysmenorrhea  ↓ NMPP  ↓ opioid use  ↑ QoL | Mild BMD loss, headache, hot flashes | (Giudice et al. 2022) |
| **Linzagolix** | Randomized controlled trial  (n=328) | 75 mg/day  Oral | ↓ pelvic pain,  ↓ dysmenorrhea,  ↓ dyschezia, well tolerated | Headache, hot flashes,  ↓ BMD, bleeding | (Donnez et al. 2020) |
|  | Phase 3 randomized controlled trial  (n=484) | 200 mg  +  ABT  Oral | ↓ pelvic pain,  ↓ dysmenorrhea,  ↓ hot flashes,  ↓ bleeding, ↑ QoL | Not clinically relevant | (Donnez et al. 2024) |
|  | Double-blind randomized extension  (n=486) | 75 mg/day  Oral  or  200 mg/day  +  ABT  oral | ↓ dysmenorrhea,  ↓ NMPP, ↓ dyschezia, ↓ dyspareunia, ↓ pelvic pain, ↑ QoL | Headache, hot flushes, anemia, fatigue, nausea, mood swings, vaginal haemorrhage, ↓ BMD | (Donnez et al. 2026) |
| **Opigolix** | Phase 2 randomized controlled trial  (n=549) | 3, 5, 10 or 15 mg/day  Oral  +  3,75 mg/month Subcutaneous | ↓ pelvic pain,  ↓ general pain,  ↓ NMPP,  ↓ dysmenorrhea | Insomnia, headaches,  tinnitus, hot flashes,  gastrointestinal problems | (D’Hooghe et al. 2019) |

*↑* increase; ↓ decrease; *ABT, add-back therapy; BMD,* bone mineral density; *COC, combined oral contraceptive; QoL,* quality of life; *NMPP,* non-menstrual pelvic pain.

| **Table S4. Selective receptor modulators used in EM treatment.** | | | | | |
| --- | --- | --- | --- | --- | --- |
| **Selective estrogen modulators used in EM treatment.** | | | | | |
| **Drug (class)** | **Study design and participants** | **Dosage and route of administration** | **Beneficial effects** | **Side effects** | **References** |
| **Bazedoxifene** | Case series  (n=5) | 20 mg/day  +  CE + leuprolide  6 months | ↓ menstrual flow and duration,  ↓ pelvic pain,  ↓ lesion size | Not described | (Hill et al. 2018) |
| **Selective progesterone receptor modulators used in EM treatment.** | | | | | |
| **Drug (class)** | **Study design and participants** | **Dosage and route of administration** | **Beneficial effects** | **Side effects** | **References** |
| **Mifepristone** | Randomized controlled trial  (n=150) | 12.5 mg/day  +  gestrinone  Oral  6 months | ↓ dysmenorrhea,  ↓ dyspareunia, ↓ pelvic pain,  ↓ pelvic tenderness | Not described | (Xue et al. 2016) |
|  | Retrospective observational study  (n=11) | (5–25 mg/day)  Oral  Long-term | Not evaluated | Morphological changes mimicking borderline endometrioid tumors; no evidence of malignant transformation | (Pan et al. 2022) |
| **Ulipristal acetate** | Case report  (n=1) | 15 mg/day  Oral  3 months | ↓ inflammation,  ↓ pelvic pain | Not described | (Bressler et al. 2017) |

↓ decrease; *CE,* conjugated estrogens.

| **Table S5.** **Aromatase inhibitors used in EM treatment.** | | | | | |
| --- | --- | --- | --- | --- | --- |
| **Drug (class)** | **Study design and participants** | **Dosage and route of administration** | **Beneficial effects** | **Side effects** | **References** |
| **Anastrozole** | Randomized clinical trial  (n=31) | 1 mg/day  Oral  6 months | ↓ dysmenorrhea,  ↓ CPP, ↓dyspareunia | Not reported | (Acién et al. 2021) |
|  | Phase 2 prospective  open-label trial  (n=15) | 1 mg/day  +  COC  Oral  6 months | ↓ CPP (40–55%),  ↓ estradiol | Breakthrough bleeding, mild hypoestrogenic symptoms; no significant BMD changes | (Amsterdam et al. 2005) |
| **Letrozole** | Prospective randomized controlled trial  (n=35) | 2.5 mg/day  +  NETA  Oral  6 months | ↓ NMPP,  ↓ dyspareunia,  ↓ endometriotic nodule volume | ↑ weight, arthralgia, intermenstrual bleeding, depression,  ↓ libido | (Ferrero et al. 2011) |
|  | Prospective observational study  (n=64) | 2.5 mg/day  +  COC  Oral  6 months | ↓ CPP, ↓ deep dyspareunia; greater pain reduction than COC alone | Migraine, arthralgia, myalgia, hair loss; mild adverse events | (Zhao et al. 2021) |

↓ decrease; *BMD,* bone mineral density; *COC,* combined oral contraceptive; *CPP,* chronic pelvic pain*; NMPP,* non- menstrual pelvic pain; *NETA, norethisterone acetate.*
